# The *tilt* ( *tt* ) mutation of *Drosophila melanogaster* maps to the cis-regulatory region of the Iroquois Complex (Iro-C) located in the *sosondowah* ( *sowah)* gene

**DOI:** 10.17912/micropub.biology.001704

**Published:** 2025-07-16

**Authors:** Samuel Gruber, Arno Houtman, Hailey Reisert, Mina Amini, Caroline Fiore, Paula Gonzalez, Veronica Han, Aeva Jazic, Mie Kusupholnand, Max Miller, Jiung Nam, Ziqin Wang, Yang Yu, Peter Dong, Allen S. W. Oak, Arun Sharma, Eric P Spana

**Affiliations:** 1 Department of Biology, Duke University, Durham, North Carolina, United States; 2 Duke Kunshan University, Kunshan, Jiangsu, China; 3 Department of Dermatology, Perelman School of Medicine at the University of Pennsylvania, Philadelphia, Pennsylvania, United States; 4 Department of Biomedical Sciences; Board of Governors Regenerative Medicine Institute; and Smidt Heart Institute, Cedars-Sinai Medical Center, Los Angeles, California, United States

## Abstract

The *tilt* ( *tt* ) mutation first described by Morgan and Bridges in 1915 has adult visible phenotypes in wing posture and vein formation. We have mapped *tt* to a genomic region within the *sosondowah* ( *sowah* ) gene that houses the cis-regulatory elements that control expression of the Iroquois Complex genes *araucan* ( *ara* ) and *caup olican* ( *caup* ) in the wing hinge and wing veins. Sequence analysis of the *tt ^1^* allele has identified a gtwin retrotransposon containing su(Hw) insulator binding sites within this region of *sowah* . We find that mutations in *su(Hw)* suppress the *tilt* phenotype, providing a potential mechanism for the insertion to affect the function of the Iroquois Complex.

**Figure 1. Mapping the tilt ( tt ) mutation:**
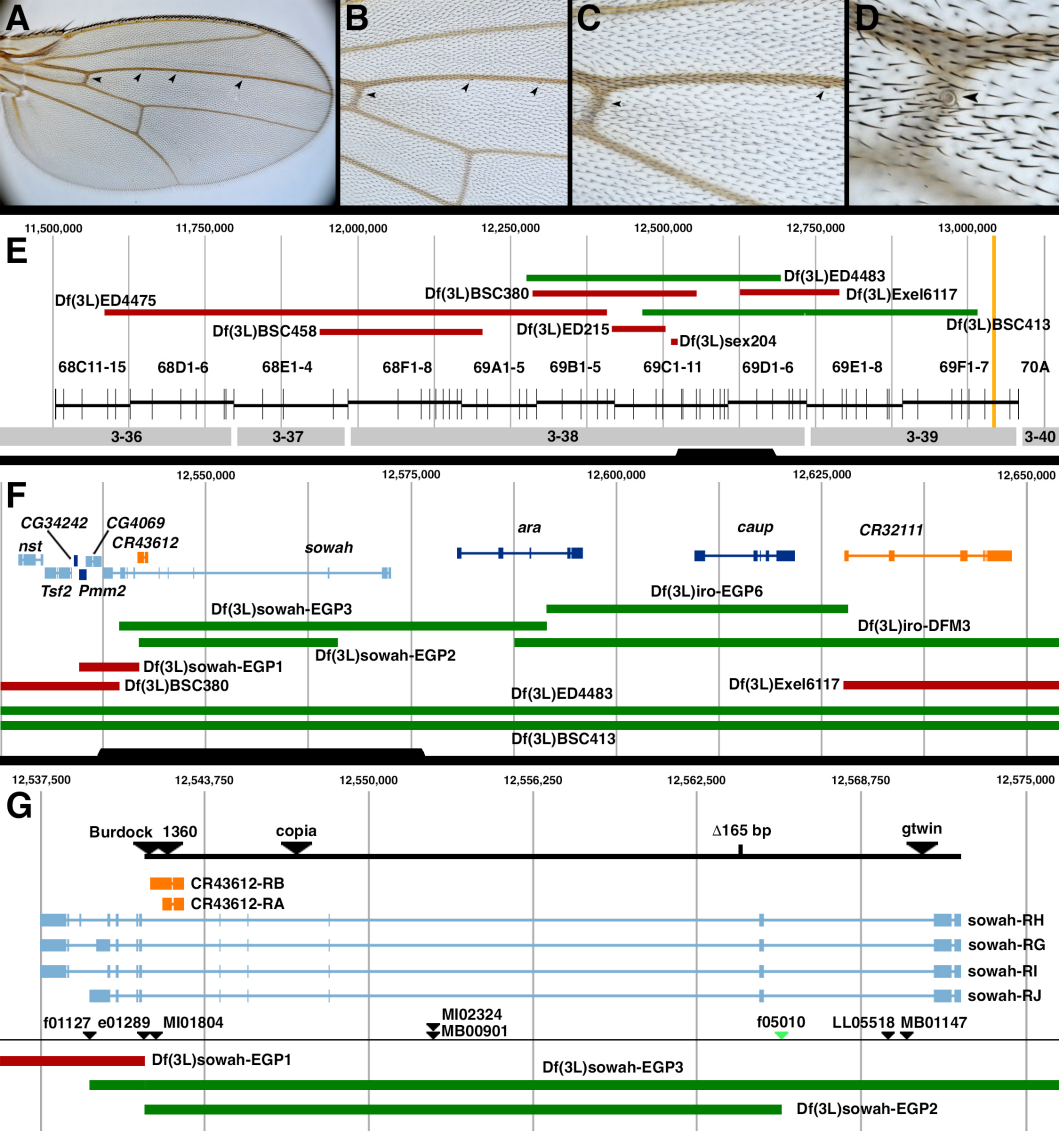
**A.** A 100X magnification of a wild-type (Oregon R) male wing. The location of the campaniform sensilla at the anterior crossvein (ACV) and the three sensilla on vein 3 (L3-1, L3-2, L3-3) are indicated with arrowheads. **B.** A 200X magnification of the same wing as A. The ACV, L3-1, and L3-2 sensilla are indicated with arrowheads. **C.** A 400X magnification of the same wing as A. The ACV and L3-1 sensilla are indicated with arrowheads. The ACV is difficult to see, as it is unpigmented. **D.** A 1000X magnification of the same wing as A. The ACV is indicated with an arrowhead. **E.** The molecular map of a 1.625 Mb region of 3L between 68C and 70A. The green bars indicate deficiency regions that fail to complement *tilt* , while red bars are regions that complement *tilt* . The yellow vertical line on the right is the location of *RpS4* . Under the deficiency regions are the cytological divisions, and below that in gray are the calculated genetic map positions in the area. The enlarged region of the black bar is the region expanded in F. **F.** The molecular map of a 125 Kb region of 3L with its ten genes. Large bars indicate exons while the connecting lines indicate introns. Orange genes are non-protein-coding transcripts transcribed left to right. Blue genes are protein-coding genes transcribed left to right (dark blue), or right to left (light blue). Deficiency regions are shown as described in E and deficiencies that extend past the edges of the image are simply shown as ending at the edge. The enlarged region of the black bar is the region expanded in G. **G.** The molecular map of a ~40 Kb region of 3L. Four transcripts of the *sowah* gene are shown as well as the non-coding *CR43612* . The thick black bar indicates the region of detailed genome sequence analysis. The large black triangles indicate the four retrotransposons found in the *tilt* genome, and the small vertical line indicates the position of a 165 bp deletion. The thin black line with small triangles indicates the position of transgene insertions tested for complementation. Only one, *sowah ^f05010^* , failed to complement *tilt* (in green). The Deficiency region colors are shown as described in E.

## Description

Mutations that changed the size, location, or presence of wing veins have been identified since the dawn of *Drosophila* genetics (Bridges & Morgan, 1919, 1923; Morgan & Bridges, 1916). These mutations have been instrumental in establishing the formation and differentiation of wing veins as a model for developmental patterning. Vein 3 is essentially established by the *hedgehog* ( *hh* ) and *decapentaplegic* ( *dpp* ) signal transduction pathways which activate the expression of a set of transcription factors of the Iroquois Complex (Iro-C) (Gomez-Skarmeta et al., 1996). These pro-veins are then refined by the *dpp* , *EGF Receptor* , and *Notch* signaling pathways (Tripathi & Irvine, 2022). A loss of vein phenotype can happen in multiple steps of the vein specification pathway from wing disc patterning, pre-patterning veins, establishing pro-veins, and finally veins.

The *tilt* mutation was identified by Calvin Bridges in August 1915 and mapped to 0.2 map units to the left of *Dichaete* (Bridges & Morgan, 1923). The *tilt* phenotype was described as having wings that were held out and up from the body and a loss of a portion of vein 3. In addition to the loss of vein, *tilt* mutant wings never had the full complement of the campaniform sensilla normally found on vein 3 (Thompson Jr. et al., 1982). A recent paper (Houtman et al., 2023) describes in detail the three observable phenotypes found in adult *tilt ^1^* flies: vein loss, sensilla loss, and wing posture defects. Of these three, only the sensilla loss phenotype is penetrant enough to accurately score *tilt* via a complementation test. One early experiment in mapping *tilt* placed it to the left (distal) of *Minute-h* (Mossige, 1938) which is now called *M(3)69E* , and more recent analysis has identified *M(3)69E* as *RpS4* (Marygold et al., 2007). Because we now had a phenotype that could be accurately scored, and a more defined genetic location, we proceeded to map the *tilt* mutation in an undergraduate lab course. We elected to focus our mapping to the left of *RpS4* (yellow line in Figure 1E ) and selected deficiency strains between 68C and 69F to test complementation by sensilla loss.

We selected eight deficiency strains and tested complementation with *tilt ^1^* in mounted wings. Because the position of the sensilla can vary in *tilt* mutants (Houtman et al., 2023), we counted the anterior crossvein sensilla (ACV) and the total number of the three sensilla on vein 3 (L3-1, L3-2, L3-3) for each wing. Figure 1A- D shows the location of these four sensilla in a wild-type OreR wing at increasing magnifications. We found six deficiency strains complemented *tilt ^1^* (visualized in Figure 1E in red): Df(3L)ED4475 (n=10, ACV=10/10, L3=32/30); Df(3L)BSC458 (n=20, ACV=20/20, L3=60/60); Df(3L)BSC380 (n=19, ACV=19/19, L3=58/57); Df(3L)ED215 (n=10, ACV=10/10, L3=32/30); Df(3L)Sex204 (n=14, ACV=14/14, L3=45/42); and Df(3L)Exel6117 (n=17, ACV=17, L3=52/51) where n is the number of wings counted, and ACV and L3 are the number of sensilla observed over the expected. Wild-type wings (OregonR) always has an ACV, and will occasionally have a fourth sensilla on vein 3, but never has fewer than three–indicating that wild-type typically has more than expected L3 sensilla (n=56, ACV=56, L3=169/168). We also found two deficiency strains that failed to complement: Df(3)ED4483 (n=21, ACV=7/21, L3=24/63); and Df(3L)BSC413 (n=12, ACV=0/12, L3=6/36) visualized in Figure 1E in green. The results for the non-complementing deficiencies were similar to *tilt ^1^* homozygotes (n=61, ACV=4/61, L3=45/183). These crosses place the *tilt* locus between the right breakpoint of Df(3L)BSC380 and the left breakpoint of Df(3L)Exel6117.

Figure 1F shows a higher resolution map of the region that, when deleted, fails to complement *tilt* . Within this region are three protein coding genes: *sosondowah* ( *sowah* ), *araucan* ( *ara* ), and *caup olican* ( *caup* ). The transcription factors *ara* and *caup* are redundant homeodomain proteins of the Iroquois Complex which help specify cell fates in many tissues in *Drosophila* development, including veins 3 and 5 in the wing (Gomez-Skarmeta et al., 1996). *sowah* is a predicted cell adhesion protein whose large introns house the regulatory regions that drive *ara* and *caup* expression in vein 3 (Maeso et al., 2012). We obtained smaller deficiencies throughout the *sowah* , *ara* , and *caup* genes, and tested their complementation with *tilt* by examining mounted wings and scoring in the same way ( Figure 1F ). We found that Df(3L)sowah-EGP1 complemented *tilt* (n=19, ACV=19/19, L3=58/57). Df(3L)sowah-EGP1 deletes the 3' end of sowah, and completely removes CG4096, Pmm2, and CG34242. This indicates that *tilt* is not a loss of function allele of *sowah* . We found that deficiencies that removed the transcripts of both *ara* and *caup* failed to complement *tilt* : first, Df(3L)iro-EGP6 (n=18, ACV=7/18, L3=3/54) deletes the 3' end of *ara* and all of *caup* and second, Df(3L)iro-DMF3 (n=23, ACV=7/23, L3=23/69) that deletes about half of *ara* and all of *caup* and extends off the map for ~44 kb. Two deficiencies that remove regions of *ara/ caup* regulatory regions also fail to complement *tilt* : first, Df(3L)sowah-EGP3 (n=17, ACV=3/17, L3=2/51) removes most of *sowah* , CR43612, and the 5' end of *ara* ; and second, Df(3L)sowah-EGP2 (n=32, ACV=3/32, L3=2/96) removes CR43612 and a large portion of sowah, but does not remove either *ara* or *caup* transcription units. Because we could rule out sowah by complementation, and because *ara* and *caup* are redundant, we hypothesized the lesion in *tilt ^1^* would be in the regulatory regions found within the *sowah* introns, which is roughly the breakpoints of Df(3L)sowah-EGP2. In addition to the loss of campaniform sensilla, the Df/ *tt ^1^* lines that fail to complement *tilt* frequently have the wing posture defect found in *tilt* homozygotes and the very rare loss of wing vein 3 as seen in Houtman et al., 2023.

Having narrowed the *tilt* locus to the regulatory region of Iro-C within of *sowah* , we performed complementation tests with eight publicly available transposon insertions. Figure 1G shows the *sowah* region that fails to complement *tilt* with the appropriate deficiency strains at the bottom. Just above the deficiencies are the positions of the transposon insertions tested. Of these eight insertions, seven fully complemented *tilt* : *sowah ^f01127^* (n=16, ACV=16/16, L3=52/48); *sowah ^e01289^* (n=10, ACV=10/10; L3=31/30); *sowah ^MI01804^* (n=10, ACV=10/10; L3=32/30); *sowah ^MI02324^* (n=10; ACV=10/10; L3=34/30); *sowah ^MB00901^* (n=9; ACV=9/9; L3=31/27); *sowah ^LL05518^* (n=10; ACV=10/10; L3=31/30); and *sowah ^MB01147^* (n=10; ACV=10/10; L3=31/30). One insertion, *sowah ^f05010^* (shown in green) failed to complement *tilt* (n=27, ACV=4/27, L3=2/81). In addition to failing to complement *tilt* by the campaniform sensilla assay, *tilt ^1^* / *sowah ^f05010^* shows a strong held-out wing phenotype exactly as shown in *tilt ^1^* homozygotes (Houtman et al., 2023).

With an identified molecular region of the *tilt ^1^* mutation due to its failure to complement *sowah ^f05010^* and Df(3L)sowah-EGP2, we attempted to identify the lesion in the *tilt* genome by using whole genome sequencing by Oxford Nanopore long-read technology. After de-novo assembly, and by aligning the *tilt ^1^* sequence reads to the *Drosophila* reference genome, we have identified a number of changes found in the *tilt ^1^* sequence that are not found in the reference genome. The thick black bar in Figure 1G shows the region between the left breakpoint of EGP2 and the transcription start site of *sowah* that was examined in detail. We found three retrotransposons within the Df(3L)sowah-EGP2 region: a Burdock element of 6,376 bp, a 1360 element of 1,474 bp, and a copia element of 5,113 bp. In addition, we found a fourth retrotransposon insertion just outside the region, a gtwin element of 6,870 bp. Finally, we found a deletion of 165 bp, 39 smaller deletions between 1 and 30 bp, 45 insertions between 1 and 22 bp, and 447 single nucleotide polymorphisms in the region shown in the bar. We did not find any changes in the *tilt ^1^* genome that might be an obvious choice to be the causative mutation.

Because the *tilt ^1^* allele was a spontaneous mutation it seems likely that it would be a single genomic lesion that would affect both hinge and vein expression of the Iroquois Complex. Any SNP or indel would be unlikely to alter expression from both the hinge and vein 3 enhancers as they are under different upstream regulatory signaling pathways (Ayala-Camargo et al., 2013; Hatini et al., 2013; Johnstone et al., 2013) (Crozatier et al., 2004). We hypothesized an alternative mechanism that might affect a larger regulatory region. The *sowah ^f05010^* transposon insertion is a PBac{WH} vector that contains a *suppressor of Hairy wing* ( *su(Hw)* ) binding site, sometimes called a gypsy insulator element (Thibault et al., 2004). This sequence binds the *su(Hw)* transcriptional insulator (Parkhurst et al., 1988), and might cause the vein 3 enhancers and wing hinge enhancers of *ara* and *caup* found in the *sowah* intron to be separated from the *ara* and *caup* promoters by putting them into different Topological Associated Domains (TADs) (Maeso et al., 2012; Peterson et al., 2021). Wing discs of *sowah ^f05010^* homozygous larvae show a loss of both *ara* and *caup* expression in vein 3 and hinge region (Maeso et al., 2012). The gtwin retrotransposon found in *tilt ^1^* is related to the gypsy retrotransposon (Kotnova et al., 2006; Ludwig & Loreto, 2007), and also contains su(Hw) binding sites and binds su(Hw) (Gerland et al., 2017). Thus, although the gtwin does not lie within the Df(3L)sowah-EGP2 region, it may keep the vein 3 and wing hinge enhancers from interacting with the *ara* and *caup* promoters. The Burdock, copia, and 1360 elements do not contain su(Hw) binding sites.

To test whether the *tilt ^1^* campaniform sensilla phenotype was related to the su(Hw) binding sites found in the gtwin retrotransposon insertion, we made flies homozygous for both *tilt ^1^* and *su(Hw) ^8^* . The *su(Hw) ^8^* allele is adult viable and has been shown to suppress gypsy insertion phenotypes in alleles of *yellow* ( *y* ), *cut* ( *ct* ), and *forked* ( *f* ) (Georgiev & Kozycina, 1996) (Guida et al., 2016; Li, Heng, 2018). In *tilt ^1^* , *su(Hw) ^8^* mounted wings, we found complete suppression of the *tilt* phenotype (n=45, ACV=45/45, L3=150/135) compared to *tilt ^1^* alone (n=61, ACV=4/61, L3=45/183) or *w;* *su(Hw) ^8^* alone (n=40, ACV=40/40, L3=123/120). In addition, we did not observe any *tilt ^1^* , *su(Hw) ^8^* flies with the wing posture defect that is found in 50% of *tilt ^1^* homozygotes (Houtman et al., 2023). This indicates that the *tilt* phenotype in both the hinge and vein is dependent on *su(Hw)* .

In summary, we have mapped the *tilt ^1^* allele to the wing enhancers for the Iroquois Complex that are found within the *sowah* gene. Though we cannot rule out that any number of mutations within the region might contribute to the phenotype, we found that the *tilt* phenotype is dependent on a functional *su(Hw)* gene, implying that the gtwin retrotransposon is the causative lesion in the *tilt* genome.

## Methods

***D. melanogaster* stocks and genetics:**

Fly stocks and crosses were maintained on cornmeal molasses agar food (Archon Scientific). Crosses were performed at 25° C, and males were used for all analyses. Adult wing imaging was done exactly as described in Houtman et al., 2023. Images shown are Z-stacks with image depths of between 21 mm ( Figure 1A ) and 11 mm ( Figure 1D ) created by Helicon Focus software.

**Whole Genome Sequencing:**

Genomic DNA was isolated using a Qiagen DNeasy Blood and Tissue Kit (#69504) and delivered to the Duke University Sequencing and Genomic Technologies Shared Resource where it was sequenced using a FLO-Min112 Flow Cell on a GridIon sequencer. Approximately 4.13 Gb of sequences were produced. Sequence was assembled using minimap2 (Li, H., 2018) and visualized using Tablet (Milne et al., 2013) and changes to the *tilt* genome were verified by eye for the ~30 kb region shown in Figure 1C . Sequence Reads deposited in SRA with a BioSample accession of SAMN41913838 : Run SRR29467516 from the Sequence Read Archive (SRA). Available at https://trace.ncbi.nlm.nih.gov/Traces/?view=run_browser&acc=SRR29467516

## Reagents

**Table:** 

Reagent Type	Designation	Identifier	Reference/ Source	Additional Information
Genetic Reagent ( *D. melanogaster* )	*tilt*	BDSC 623	Bloomington Drosophila Stock Center	Genotype: *tt ^1^ wo ^1^*
Genetic Reagent ( *D. melanogaster* )	*su(Hw)*	BDSC 1053	Bloomington Drosophila Stock Center	Genotype: *y ^2^ sc ^1^ v ^1^ ; su(Hw) ^8^*
Genetic Reagent ( *D. melanogaster* )	OreR	N/A	Lab Strain	Wild type
Genetic Reagent ( *D. melanogaster* )	Df(3L)BSC380	BDSC 24404	Bloomington Drosophila Stock Center	Genotype: * w ^1118^* ; Df(3L)BSC380/TM6C, *Sb ^1^* *cu ^1^*
Genetic Reagent ( *D. melanogaster* )	Df(3L)BSC413	BDSC 24917	Bloomington Drosophila Stock Center	Genotype: * w ^1118^* ; Df(3L)BSC413/TM6C, *Sb ^1^ cu ^1^*
Genetic Reagent ( *D. melanogaster* )	Df(3L)BSC458	BDSC 24962	Bloomington Drosophila Stock Center	Genotype: * w ^1118^* ; Df(3L)BSC458/TM6C, *Sb ^1^ cu ^1^*
Genetic Reagent ( *D. melanogaster* )	Df(3L)iro-DFM3	BDSC 36531	Bloomington Drosophila Stock Center	Genotype: Df(3L)iro-DFM3, *ara ^DFM3^ mirr ^DFM3^* /TM6B, *Tb ^1^*
Genetic Reagent ( *D. melanogaster* )	Df(3L)iro-EGP6	N/A	Sonsoles Campuzano (Universidad Autónoma de Madrid) (Carrasco-Rando et al., 2011)	Genotype: w*; Df(3L)iro-EGP6/TM3, Sb, Ser
Genetic Reagent ( *D. melanogaster* )	Df(3L)ED215	BDSC 8071	Bloomington Drosophila Stock Center	Genotype: * w ^1118^* ; Df(3L)ED215, P{3'.RS5+3.3'}ED215/TM6C, *cu ^1^ Sb ^1^*
Genetic Reagent ( *D. melanogaster* )	Df(3L)ED4475	BDSC 8069	Bloomington Drosophila Stock Center	Genotype: * w ^1118^* ; Df(3L)ED4475, P{3'.RS5+3.3'}ED4475/TM6C, *cu ^1^ Sb ^1^*
Genetic Reagent ( *D. melanogaster* )	Df(3L)ED4483	BDSC 8070	Bloomington Drosophila Stock Center	Genotype: * w ^1118^* ; Df(3L)ED4483, P{3'.RS5+3.3'}ED4483/TM6C, *cu ^1^ Sb ^1^*
Genetic Reagent ( *D. melanogaster* )	Df(3L)Exel6117	BDSC 7596	Bloomington Drosophila Stock Center	Genotype: * w ^1118^* ; Df(3L)Exel6117, P{XP-U}Exel6117/TM6B, *Tb ^1^*
Genetic Reagent ( *D. melanogaster* )	Df(3L)Sex204	BDSC 9337	Bloomington Drosophila Stock Center	Genotype: y ^1^ w* ; Df(3L)sex204/TM6C, *Sb ^1^ Tb ^1^*
Genetic Reagent ( *D. melanogaster* )	Df(3L)sowah-EGP1	N/A	Sonsoles Campuzano (Universidad Autónoma de Madrid) (Maeso et al., 2012)	Genotype: w*; Df(3L)sowah-EGP1/TM3, Sb
Genetic Reagent ( *D. melanogaster* )	Df(3L)sowah-EGP2	N/A	Sonsoles Campuzano (Universidad Autónoma de Madrid) (Maeso et al., 2012)	Genotype: w*; Df(3L)sowah-EGP2/TM3, Sb
Genetic Reagent ( *D. melanogaster* )	Df(3L)sowah-EGP3	N/A	Sonsoles Campuzano (Universidad Autónoma de Madrid) (Maeso et al., 2012)	Genotype: w*; Df(3L)sowah-EGP3/TM3, Sb
Genetic Reagent ( *D. melanogaster* )	sowah-f01127	BDSC 18412	Bloomington Drosophila Stock Center	Genotype: * w ^1118^* ; PBac{WH} *sowah ^f01127^*
Genetic Reagent ( *D. melanogaster* )	sowah-e01289	BDSC 85121	Bloomington Drosophila Stock Center	Genotype: * w ^1118^* ; PBac{RB} *sowah ^e01289^* /TM6B, *Tb ^1^*
Genetic Reagent ( *D. melanogaster* )	sowah-MI01804	BDSC 37311	Bloomington Drosophila Stock Center	Genotype: y ^1^ w* ; Mi{MIC} *sowah ^MI01804^* /TM3, *Sb ^1^ Ser ^1^*
Genetic Reagent ( *D. melanogaster* )	sowah-MI02324	BDSC 35109	Bloomington Drosophila Stock Center	Genotype: y ^1^ w* ; Mi{MIC} *sowah ^MI02324^*
Genetic Reagent ( *D. melanogaster* )	sowah-MB00901	BDSC 22927	Bloomington Drosophila Stock Center	Genotype: *y ^1^ w ^67c23^* ; Mi{ET1} *sowah ^MB00901^*
Genetic Reagent ( *D. melanogaster* )	sowah-f05010	N/A	Sonsoles Campuzano (Universidad Autónoma de Madrid) (Maeso et al., 2012)	Genotype: w*; sowah-f05010/TM6B
Genetic Reagent ( *D. melanogaster* )	sowah-LL5518	Kyoto 141612	Kyoto Drosophila Stock Center	Genotype: y* w* ; PBac{SAstopDsRed}LL05518 P{FRT( *w ^hs^* )}2A P{neoFRT}82B P{Car20y}96E/TM6B, *Tb ^1^*
Genetic Reagent ( *D. melanogaster* )	sowah-MB01147	BDSC 23001	Bloomington Drosophila Stock Center	Genotype: *y ^1^ w ^67c23^* ; Mi{ET1} *sowah ^MB01147^*
Software	Helicon Focus	N/A	https://www.heliconsoft.com/heliconsoft-products/helicon-focus	
Software	minimap2	N/A	https://github.com/lh3/minimap2/releases	
Software	Tablet	N/A	https://ics.hutton.ac.uk/tablet/	
